# Insights into cardiovascular health: Knowledge, attitudes, and practices in the Kurdistan region of Iraq

**DOI:** 10.1016/j.puhip.2025.100686

**Published:** 2025-12-11

**Authors:** Ahmed A. Mosa, Avan Saadi Saleh, Osama Abdullah M. Taher, Yousif Hameed Ramadhan, Kani Fareeq Farooq, Hajar Hassan Abdulqadir, Jodi Ramadhan Haji, Rojeen Chalabi Khalid, Abdulrahman Ardalan Mahdi, Rojeen Abdullah Hasso, Zainab Muhsin Hussein, Banaz Ameen Mosa, Ogen Ashur Yohanna, Arblina Adeeb Yousif, Ava Anwar M. Maasum, Salmas S. Ahmed, Ibrahim A. Naqid, Ameen M. Mohammad

**Affiliations:** a Department of Clinical Sciences, College of Medicine, University of Zakho, of, Zakho, Kurdistan Region, Iraq; b Azadi Cardiac Center, Azadi Teaching Hospital, of, Duhok, Kurdistan Region, Iraq; c College of Medicine, University of Duhok, of, Duhok, Kurdistan Region, Iraq; d Independent Researcher, of, Duhok, Kurdistan Region, Iraq; e College of Medicine, of, Hawler Medical University, Hawler, Kurdistan Region, Iraq; f Department of Biomedical Sciences, College of Medicine, University of Zakho, of, Zakho, Kurdistan Region, Iraq; g Department of Internal Medicine, College of Medicine, University of Duhok, of, Duhok, Kurdistan Region, Iraq

**Keywords:** Cardiovascular disease, Risk factors, Knowledge, Attitude, Practice

## Abstract

**Objectives:**

This study aimed to explores the knowledge, attitudes, and practices related to cardiovascular diseases (CVDs) risk factors among the Iraqi Kurdistan population to identify gaps and guide public health strategies for better cardiovascular health outcomes.

**Study design:**

A population-based study.

**Methods:**

This population-based study was conducted in Duhok Province and the Zakho Independent Administration within the Kurdistan Region of Iraq from April 20, 2024, to June 15, 2024. A total of 784 participants were enrolled in the study. Data collection was carried out through face-to-face interviews in various public settings using a questionnaire comprising 26 items divided into four sections: sociodemographic characteristics, knowledge, attitudes, and practices.

**Results:**

The mean age of the study participants was 32.48 ± 13.63 years. Females made up (54 %) of the sample, and more than half had university or postgraduate education. While a majority recognized smoking (92.1 %), hypertension (81.5 %), and high cholesterol (86.2 %) as significant CVD risk factors, (31.7 %) were unaware that diabetes increases CVD risk. Despite high awareness, only (29.7 %) regularly exercised, and (34.6 %) consumed fast food regularly. The mean KAP score was 20.66 ± 2.63 out of 25, which manifests that participant had good KAP scores toward cardiovascular disease risk factors. The KAP scores were significantly higher among females, married individuals, healthcare workers, non-smokers, those with a family history of CVD, and individuals who obtained their knowledge from physicians.

**Conclusions:**

This study reveals a gap between knowledge and practice regarding CVD risk factors in the Kurdistan Region. Awareness is high, but many, especially younger adults and those with lower education, fail to adopt healthy practices. The findings highlight the need for targeted interventions to promote behavioral changes.

## 1 What the study adds

• The study fills a significant gap in regional public health research by providing novel insights of Iraqi Kurdistan population about the knowledge, attitudes, and practices (KAP) related to cardiovascular diseases (CVDs).
• The study identifies a discrepancy between population awareness and behaviors regarding CVDs, highlighting the need for targeted behavioral change strategies.

## 2 Implications for policy and practice

• Policymakers should implement targeted public health interventions that extend beyond awareness campaigns, focusing on promoting behavioral changes by creating accessible exercise spaces and fostering a supportive environment for physical activity in public areas.
• Integrating cardiovascular health and disease-prevention education into schools, workplaces, and community centers can help promote healthy habits from an early age and reach a broader segment of the population.

## 3 Introduction

Cardiovascular diseases (CVDs) encompass a variety of conditions that impact the heart, blood vessels, or both. CVDs are the leading cause of mortality globally, responsible for a substantial proportion of annual deaths worldwide. Approximately (32 %) of all deaths, totaling 17.9 million people annually, are attributed to CVDs. Alarmingly, more than 75–80 % of these deaths occur in low and middle-income countries (LMICs) [1].

Significant societal transitions in developing nations have led to shifts in behavioral, social, and lifestyle patterns, which have contributed to the high prevalence of modifiable risk factors such as hypertension, diabetes mellitus, dyslipidemia, obesity, and smoking [2]. Epidemiological studies have shown that behaviors like smoking, lack of physical activity, unhealthy and irregular diets, and stress have a greater impact on health outcomes than genetic factors, quality of medical care, or environmental conditions. Addressing these harmful risk factors is crucial [3]. A study in Iraq identified non-genetic factors as the primary risk factors in patients with premature coronary artery disease [4,5].

The high prevalence of modifiable risk factors in our region is largely preventable or manageable through lifestyle changes and medical interventions, underscoring the critical need for knowledge and awareness about CVDs and their risk factors [6]. Comprehensive patient education can significantly aid in managing various health issues. Well-informed patients are more likely to adhere to prevention and management recommendations [7,8]. Knowledge and beliefs can either hinder preventive measures or promote a healthy lifestyle. Success in preventing and treating CVDs depend on understanding these conditions and their risk factors. Reducing exposure to modifiable risk factors can lower lifetime risk and foster healthy attitudes [9].

Numerous studies worldwide have extensively examined the knowledge and awareness of CVD symptoms and risk factors among patients and the general population. However, there is a notable gap in understanding within Iraq. Therefore, this research aims to explore the interplay between knowledge, attitudes, and practices related to CVD risk factors among the Iraqi Kurdistan population, and to assess the factors influencing their knowledge of these risk factors. By evaluating the population's knowledge, we can identify prevalent gaps and misconceptions that hinder the effective prevention and management of CVDs. This will guide the regional government in developing targeted educational programs and public health strategies to address these common risk factors.

## 4 Methods

### 4.1 Study Design and participants

This population-based study was conducted in the Duhok province and the Zakho independent administration of the Kurdistan Region of Iraq. Data collection took place from April 20, 2024, to June 15, 2024, during which a total of 784 participants were enrolled. The questionnaires were administered through face-to-face interviews conducted by one of the authors in various settings, including public places, hospitals, universities, family homes, and public gardens.

**Fig. 1:**
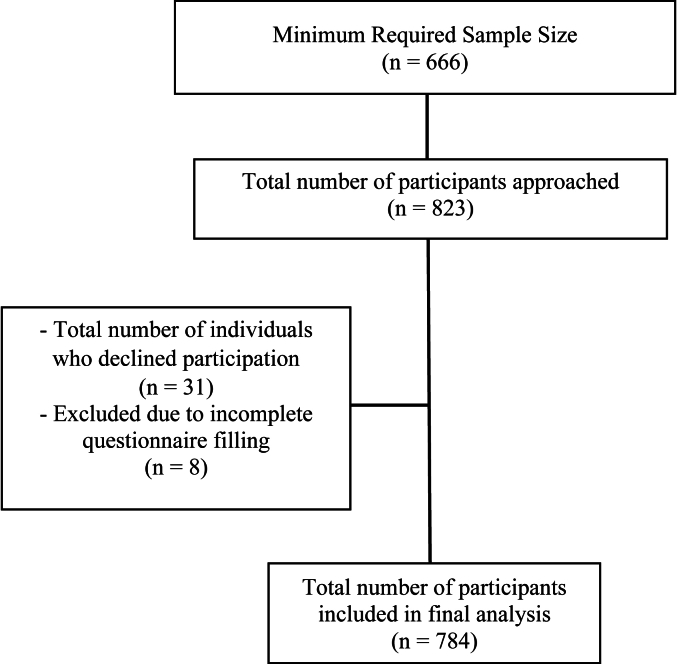
Participants recruitment flowchart.

An online sample size calculator (http://www.raosoft.com/samplesize.html) was utilized to estimate the necessary study sample size. For a population of 1.5 million, with a confidence level of 99 %, a margin of error of 5 %, and a population proportion of 50 %, the minimum required sample size was determined to be 666 participants. Fig. 1 illustrates flowchart of participant enrolment.

### 4.2 Data collection

To ensure representative sampling of the population, a multistage sampling method was employed. The city was divided into several clusters, including public places, hospitals, universities, and residential areas, with each cluster representing a distinct segment of the region's population. The research team was divided into multiple groups, each responsible for data collection within a specific cluster. To minimize potential bias and enhance representativeness, participants were randomly selected within each cluster. Regionally, approximately (65 %) of individuals aged 18 years and above fall within the (18–39 years) age group. This indicates that the study sample closely reflects the regional demographic distribution, with only a slight overrepresentation of the (18–39 years) age group.

### 4.3 Study measurements

The study questionnaire was adapted from a previously validated cross-sectional study, with modifications made by the author to better suit the regional participants [9]. The final version of the survey was evaluated by two experts in the field of cardiology and public health to ensure the comprehensiveness and content validity of the questionnaire. A pilot testing was then conducted on 20 individuals from general population to assess clarity, understanding and time required for completion. The questionnaire comprised 26 items designed to assess the population's knowledge, attitude, and practice (KAP) regarding cardiovascular disease risk factors. The survey was organized into four sections: sociodemographic characteristics, knowledge, attitude, and practice.

#### 4.3.1 Section I - sociodemographic characteristics

This section included nine items to capture the sociodemographic characteristics of the study participants. Questions addressed age, gender, marital status, academic level, residency, smoking status, past medical history, family history, and sources of cardiovascular disease knowledge.

#### 4.3.2 Section II - knowledge

This section contained eight multiple-choice questions evaluating the participants' knowledge of cardiovascular disease risk factors. Participants were required to answer "yes," "no," or "I don't know" to each of the eight items. A score of 1 was assigned for each "yes" response, while "no" and "I don't know" responses received a score of 0. The total score for the knowledge section ranged from 0 to 8.

#### 4.3.3 Section III - attitude

This section of the study possessed the questions to determine the attitude of the participants toward a healthier lifestyle. The questions in the attitude section were evaluated by a 3-point Likert scale. Ranging from 1 to 3 from disagree to agree. The total score for the attitude section ranged from 4 to 12.

#### 4.3.4 Section IV - practice

In the practice section, participants answered five questions related to their behaviors and practices concerning cardiovascular disease risk factors. Participants responded "yes" or "no" to each statement. A "yes" response scored 1 point, while a "no" response scored 0 points. However, two questions in this section (“Do you experience sleep disturbances" and "Do you eat fast food regularly") were reversely scored. The total score for this section ranged from 0 to 5.

The maximum total score for the knowledge, attitude, and practice sections ranged from 4 to 25.

### 4.4 Inclusion/exclusion criteria

Participants aged 18 and older who consented to participate were included in the study. However, those younger than 18 and any questionnaires with incomplete information or missing data were excluded from the study.

### 4.5 Ethical consideration

The Ethics Committee at the College of Medicine, University of Zakho, Kurdistan Region of Iraq provided formal approval for the final study questionnaire on March 18, 2024, with a reference number of (MAR2024/E03). All study participants provided informed consent. Participants' anonymity was maintained, and their participation was entirely voluntary. The study adhered to the Ethical Standards for Medical Research Involving Human Subjects as outlined in the Declaration of Helsinki.

### 4.6 Statistical analysis

The data were cleaned and coded, and statistical analysis was performed using GraphPad Prism and Microsoft Excel software. The categorical variables of participants were described using frequencies and percentages, while continuous variables were evaluated using means, standard deviations, and medians. To determine the association between sociodemographic characteristics and total KAP score, unpaired two-tailed t-tests and ordinary one-way ANOVA tests were conducted. An association was considered significant if the p-value was less than 0.05.

## 5 Results

### 5.1 Basic demographic characteristics

**Fig. 2:**
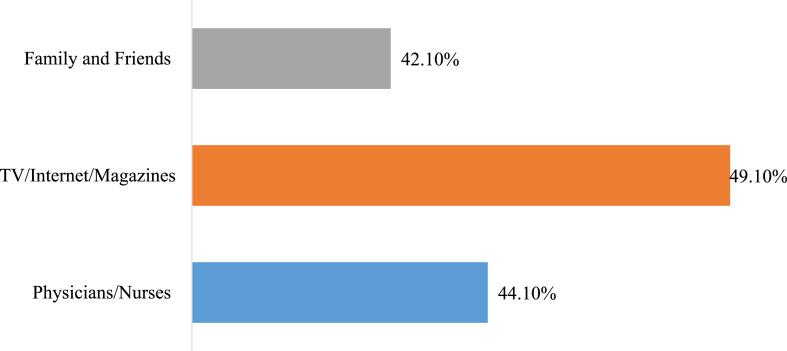
Sources of cardiovascular disease knowledge.

The mean age of the study participants was 32.48 ± 13.63 years, with approximately three-quarters of the participants falling within the 18–39 age range. More than half of the participants were female, and 454 (57.9 %) of the participants were single. Approximately half of the participants possessed university degrees or postgraduate qualifications, and 59 (7.5 %) were healthcare workers. Out of the total, 449 (57.3 %) resided in Duhok province. About two-thirds of the individuals were non-smokers. Regarding the medical history of cardiovascular disease (CVD) risk factors, about three quarters of the sample reported no history of diseases such as diabetes mellitus, hypertension, ischemic heart disease, high cholesterol, or other relevant conditions. In terms of family history of CVD, 384 (49 %) of participants reported a positive family history, while 400 (51 %) reported a negative family history. Table 1 provides a detailed summary of the sociodemographic characteristics of the participants. Fig. 2 shows sources of cardiovascular disease knowledge among the participants.

**Table 1:** Sociodemographic characteristics of the participants (n = 784).

Variables	n (%)
**Age**	
Mean (SD)	32.48 (±13.63)
18-39	585 (74.62)
40-59	154 (19.64)
>59	45 (5.74)
**Gender**	
Male	361 (46)
Female	423(54)
**Marital status**	
Single	454 (57.9)
Married	314 (40.1)
Divorced or widowed	16 (2)
**Academic Level**	
Illiterate/Primary education	131 (16.7)
Middle school/High school	185 (23.6)
University/Postgraduate	409 (52.2)
Health-care workers	59 (7.5)
**Residency**	
Duhok	449 (57.3)
Zakho	169 (21.6)
Others	166 (21.2)
**Smoking Status**	
Smoker	200 (25.5)
Non-smoker	534 (68.1)
Ex-smoker	50 (6.4)
**Medical history**	
Diabetes Mellitus	39 (5)
Hypertension	75 (9.6)
Ischemic heart disease	27 (3.4)
High Cholesterol	64 (8.2)
None	611 (77.9)
Others	51 (6.5)
**Family History of cardiovascular disease**	
Yes	384 (49)
No	400 (51)

### 5.2 Knowledge about cardiovascular risk factors

Table 2 presents the participants' knowledge regarding CVD risk factors. A substantial majority, 722 participants (92.1 %), were aware that smoking and obesity can lead to heart and blood vessel disease. Notably, about one-third of the participants were unaware that diabetes mellitus increases the risk of CVD. Conversely, a significant proportion 639 participants (81.5 %) recognized hypertension as a risk factor for cardiovascular disease. Furthermore, 676 participants (86.2 %) understood that high cholesterol levels elevate the risk of CVD. Regarding the role of exercise in preventing heart and blood vessel disease, 85 individuals (10.8 %) were unaware of its benefits. In contrast, approximately 672 participants (85.7 %) acknowledged that consuming fast food increases the risk of CVD. Additionally, 115 participants (14.6 %) were not aware that cardiovascular disease can affect young individuals. A considerable majority, 697 individuals (88.9 %), identified high stress as a risk factor for CVD.

**Table 2:** Participants' knowledge about cardiovascular diseases (n = 784).

Variables	Correct answer n (%)
Smoking and Obesity can cause heart and blood vessel disease	722 (92.1)
Having DM increases the risk of heart and blood vessel	536 (68.4)
Having HTN increases the risk of heart and blood vessel disease	639 (81.5)
Having high cholesterol can increase the risk of CVD	676 (86.2)
Adequate exercise can prevent heart and blood vessel disease	699 (89.2)
Eating fast food increases the risk of heart and blood vessel disease	672 (85.7)
CVD can occur in young people	669 (85.3)
High stress in life can cause heart and blood vessel disease	697 (88.9)

DM: Diabetes Mellitus; HTN: Hypertension; CVD: Cardiovascular disease.

### 5.3 Participant's attitude toward a healthy lifestyle

**Fig. 3:**
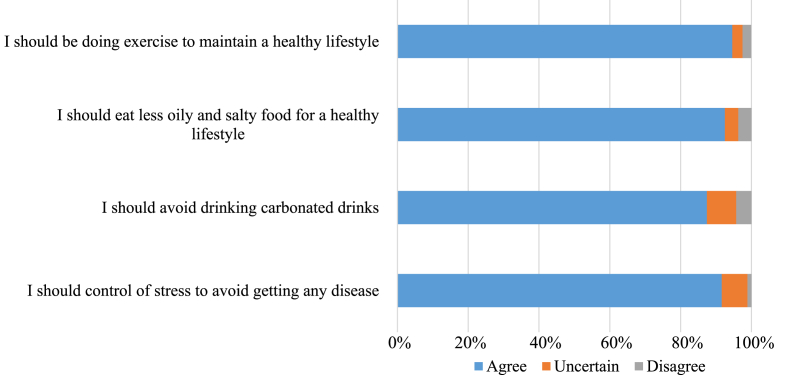
Participants' attitude towards cardiovascular diseases (n = 784).

Fig. 3 illustrates the participants' attitudes toward CVD risk factors. A vast majority, 742 participants (94.6 %), agreed that engaging in regular exercise is essential for maintaining a healthy lifestyle. Over ninety percent of the study cohort concurred that reducing the consumption of oily and salty foods is necessary. Additionally, 685 participants (87.4 %) agreed on the importance of avoiding carbonated drinks, while only 65 participants (8.3 %) expressed uncertainty regarding this. Approximately nine-tenths of the participants affirmed the necessity of managing stress to prevent cardiovascular diseases.

### 5.4 Practices for preventing cardiovascular diseases

**Fig. 4:**
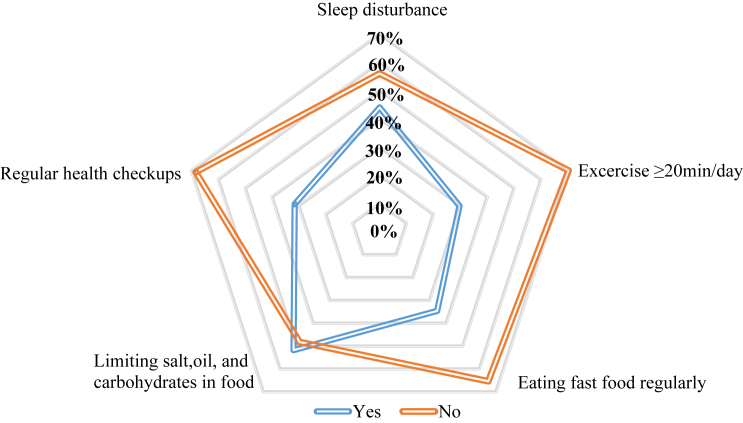
Participants practice towards cardiovascular diseases (n = 784).

Fig. 4 shows the practice of participants towards cardiovascular diseases. About 345 (44 %) of responders experienced sleep disturbance. Unfortunately, about two-thirds of the participants didn't spend at least 20 min per day to perform exercise. Surprisingly, one-third reported eating fast food regularly. Nearly half of responders limited amounts of salt, oil, and carbohydrates in their food. Approximately, two-thirds of participants didn't go to the doctor for health checkups.

### 5.5 Mean knowledge, attitude, and practice score and correlation with sociodemographic characteristics

Table 3 presents the summation scores of knowledges, attitude, and practice (KAP). While the maximum possible KAP score was 25, the mean KAP score among our study participants was 20.66 ± 2.63. Table 4 illustrates the comparison of KAP scores with participants’ sociodemographic characteristics. Notably, the mean KAP score was significantly higher in individuals aged 40–59 years compared to other age groups, with a p-value of (<0.0001). Females exhibited better KAP score, with a statistically significant p-value of (0.0015). Married participants and healthcare workers had mean KAP scores of 21.21 ± 2.45 and 21.85 ± 1.87, respectively, which were the highest within their respective categories. Additionally, participants residing in Zakho and non-smokers demonstrated superior KAP scores with significant p-values. Participants with a history of ischemic heart disease and those with a family history of CVD achieved the highest KAP scores within their categories. However, the variable of past medical history was the only variable missing the statistical significance.

**Table 3:** Summation of knowledge, attitude, and practice score (n = 784).

Variables	Maximum possible score	Mean	Standard deviation	Median
Knowledge score	8	6.77	1.49	7
Attitude score	12	11.54	0.98	12
Practice score	5	2.35	1.27	2
Total score	25	20.66	2.63	21

**Table 4:** Comparison of the participant's sociodemographic characteristics and KAP score (n = 784).

Variables	Mean KAP score	Standard deviation	p-value
**Age**			
18-39	20.39	2.7	<0.0001
40-59	21.62	2.16	
>59	20.98	2.29	
**Gender**			
Male	20.34	2.99	0.0015
Female	20.94	2.24	
**Marital status**			
Single	20.29	2.67	<0.0001
Married	21.21	2.45	
Divorced or widowed	20.5	2.83	
**Academic Level**			
Illiterate/Primary education	20.47	2.47	0.0004
Middle school/High school	20.23	2.82	
University/Postgraduate	20.75	2.63	
Health-care workers	21.85	1.87	
**Residency**			
Duhok	20.44	2.66	<0.0001
Zakho	21.46	2.26	
Others	20.45	2.76	
**Smoking Status**			
Smoker	19.83	3.11	<0.0001
Non-smoker	21.03	2.31	
Ex-smoker	20.06	2.91	
**Medical history**			
Diabetes Mellitus	21.15	2.48	0.089
Hypertension	21.09	2.35	
Ischemic heart disease	21.63	2.95	
High Cholesterol	20.19	2.97	
None	20.60	2.64	
Others	20.84	2.58	
**Family History of cardiovascular disease**			
Yes	20.96	2.48	0.0021
No	20.38	2.74	
**Sources of Cardiovascular disease knowledge**			
Family and friends	19.91	2.45	<0.0001
TV/Internet/Magazines	20.34	2.65	
Physician/Nurse	21.23	2.45	
Combination of ≥2 sources	20.9	2.78	

## 6 Discussion

Cardiovascular diseases (CVDs) are the leading cause of death globally, as reported by the World Health Organization (WHO). Notably, many CVDs can be prevented by addressing key behavioral and environmental risk factors such as tobacco use, unhealthy diets, obesity, physical inactivity, and excessive alcohol consumption. The present study aims to assess population knowledge, attitude, and practice towards cardiovascular diseases in Duhok province, Kurdistan Region of Iraq. The study participants revealed very good level of knowledge and positive attitudes towards CVDs. In contrast, they demonstrated a very low level of healthy practices, which is the most crucial aspect for prevention of CVDs. These findings will help us to assess the effectiveness of current community education programs and guide for future public health campaigns aiming at enhancing population knowledge, attitudes, and particularly practices of CVDs prevention.

The current study identified that TV, internet, and magazines as the most common sources utilized by population for information about CVDs. This aligns with a previous study conducted in UAE [9], reinforcing the significance of these platforms in raising public awareness and their role as primary source for sharing information on various subjects, highlighting their importance in shaping public knowledge and perceptions. The vast majority of the study participants were aware that smoking and obesity are risk factors for CVDs, with over (90 %) of respondents identifying them as key contributors. Majority of them also stated that unhealthy diet, physical inactivity, and stress are risk factors. These findings are consistent with results from previous studies conducted in Saudi Arabia, Jordan, and Kuwait [6,10,11]. The participants' high level of knowledge regarding these risk factors can be attributed to awareness initiatives and campaigns disseminated through mass media.

However, about one-third of the participants were not aware that diabetes mellitus is a major risk factor for cardiovascular diseases. This finding is not unique to our study as a lower percentage of participants from Saudi Arabia and Kuwait recognized diabetes mellitus as a risk factor [6,11]. In contrast a study from Tehran, Iran reported that about (80 %) of their population are aware about the harms of diabetes mellitus [12]. This highlights the necessity of enhancing media coverage and implementing public awareness campaigns among both diabetic patients and the general population, emphasizing that diabetes significantly increases the risk of developing cardiovascular diseases in the future. Contrarily, and fortunately, our respondents demonstrated a better awareness of hypertension and elevated cholesterol level as significant risk factors for cardiovascular diseases.

Although (94.6 %) of the respondents expressed willingness to exercise regularly, indicating a highly positive attitude toward physical activity, only (30 %) reported actually exercising for at least 20 min per day. Similarly, while over (90 %) agreed on the necessity of reducing the consumption of oily and salty foods as well as carbonated drinks, only half adhered to such dietary restrictions, and one-third consumed fast food regularly. These results align with studies carried out in Lebanon and Iran, which also revealed a significant gap between awareness and actual behavior [[12], [13], [14]].

This trend of sedentary lifestyles is very common among Middle Eastern populations and is highly concerning. According to a report, the Middle East has one of the worst physical activity profiles globally [15], also physical inactivity, along with unhealthy diets, are among the most common CVD risk factors across nearly all countries in this region [16]. Several studies on coronary artery diseases (CADs) in Kurdistan Region of Iraq have reported these risk factors as a major contributor for development of CADs among their population. Therefore, the government should focus on specialized programs and campaigns to increase healthy practice among their population [[17], [18], [19], [20]].

In the present study, about (90 %) of the study population were aware about the importance of controlling their stress, and its role in protecting them from getting the diseases. This aligns with a study conducted in UAE, in which (95 %) of their participants stated that they should control their stress [9]. Despite acknowledging the importance of stress management by study participants, about half of them admitted experiencing sleep disturbances. This finding is particularly important given the well-established correlation between sleep disruption and increased stress levels [21].

The overall average of correct answers in knowledge section was (84.63 %), expressing a very good knowledge level among study participants. Studies from Saudi Arabia and Turkey reported a lower overall average of correct answers which was (72.15 %) and (69.64 %), respectively [22,23]. The current study population exhibited an excellent attitude toward CVDs prevention, this finding is crucial, as a positive attitude is linked to the motivation towards healthier behaviors, which is essential in the prevention of CVDs. According to behavioral models, individuals with a better attitude are more likely to be motivated to make healthier lifestyle changes [24]. In contrast to present study, the Iranian population reported a moderate prevention attitude towards CVDs [12]. Regarding the scope of practices, our participants had low mean score of a healthy practice, in which the overall percentage of health practice was (53 %). In comparison, a study conducted in Sulaimani city, Iraqi Kurdistan reported a very low overall percent of healthy practice [25]. Both studies exhibiting a significant gap between population attitude and practice of healthy behavior. A study from Lebanon reported a high percent (70 %) of healthy practice among their population [13].

On analyzing the sociodemographic variables relation with the total KAP score, certain variables demonstrated better KAP score compared to their counterparts. We found that participants aged between 40 and 59 years have better KAP score. This result contrasts studies conducted in Kuwait and Saudi Arabia which reported that younger individuals less than 40 years of age have better KAP score [11,23]. This difference may be caused by variations in study populations, access to education, and the availability of resources like the internet. In terms of gender, female demonstrated a better KAP score when compared to male gender, finding similar to studies carried out in neighboring countries such as Jordan, Kuwait, and Turkey [10,11,22]. This may be attributed to females being more attentive and concerned about health issues, particularly in our region. Individuals with a positive family history of cardiovascular diseases exhibited higher KAP score. This finding aligns with the results from studies conducted in Jordan and Turkey [10,22].

### 6.1 Strengths and limitations

The present study stands as one of the few studies conducted about cardiovascular diseases knowledge, attitude, and practice (KAP) in our region, with a large sample size which will enable us to generalize the results to the Duhok province, Iraqi Kurdistan. This study will serve as a valuable resource in shaping educational initiatives and public health strategies to effectively address these prevalent risk factors. Despite this the study has several limitations; first cross-sectional design of study limits our ability to draw conclusions about causality or to track changes in KAP over time. Secondly, the study survey did not comprehensively assess all areas of KAP. Finally, the study was carried out only in one province of Iraqi Kurdistan, Further studies are required to assess population KAP in other provinces. However, the research will play an important role in future research about this topic in region.

### 6.2 Conclusions and recommendations

This study highlights a significant discrepancy between knowledge and behavior concerning CVDs risk factors in the Kurdistan Region of Iraq. While there is considerable awareness of major CVDs risk factors, including smoking, hypertension, and high cholesterol, many individuals, particularly younger adults and those with lower levels of education, do not implement healthy lifestyle behaviours. The findings emphasize the need for targeted public health initiatives that not only raise awareness but also encourage behavioral modifications, particularly among high-risk groups such as smokers and individuals with a family history of CVDs. Addressing these issues could help reduce the prevalence and impact of CVDs, ultimately improving public health outcomes in the region.

## Data availability statement

All data supporting the findings of this manuscript are available without restriction.

## Authors’ contribution statement

All authors have substantially contributed in data collection, analysis, manuscript writing and

approved the final version of manuscript to be submitted.

## Ethical statement

The Ethics Committee at the College of Medicine, University of Zakho, Kurdistan Region of Iraq provided formal approval for the final study questionnaire on March 18, 2024, with a reference number of (MAR2024/E03). All study participants provided informed consent. Participants' anonymity was maintained, and their participation was entirely voluntary.

## Declaration of generative AI and AI-assisted technologies in the writing process

During the preparation of this work the authors used ChatGPT 4o in order to improve the linguistic format of the manuscript. After using this tool, the authors reviewed and edited the content as needed and takes full responsibility for the content of the published article.

## Funding

This research did not receive any specific grant from funding agencies in the public, commercial, or not-for-profit sectors.

## Declaration of competing interest

The authors declare that they have no known competing financial interests or personal relationships that could have appeared to influence the work reported in this paper.
